# Intraspecific Leaf Trait Variation across and within Five Common Wine Grape Varieties

**DOI:** 10.3390/plants11202792

**Published:** 2022-10-21

**Authors:** Samantha C. Macklin, Rachel O. Mariani, Emily N. Young, Rosalyn Kish, Kimberley A. Cathline, Gavin Robertson, Adam R. Martin

**Affiliations:** 1 Department of Physical and Environmental Sciences, University of Toronto Scarborough, Toronto, ON M1C 1A4, Canada; 2 Horticultural & Environmental Sciences Innovation Centre, Niagara College, Niagara-on-the-Lake, ON L0S 1J0, Canada

**Keywords:** agroecology, functional trait, intraspecific trait variation, Leaf Economics Spectrum, plant trait spectra, *Vitis vinifera*

## Abstract

Variability in traits forming the Leaf Economics Spectrum (LES) among and within crop species plays a key role in governing agroecosystem processes. However, studies evaluating the extent, causes, and consequences of within-species variation in LES traits for some of the world’s most common crops remain limited. This study quantified variations in nine leaf traits measured across 90 vines of five common wine grape (*Vitis vinifera* L.) varieties at two growth stages (post-flowering and veraison). Grape traits in these varieties covary along an intraspecific LES, in patterns similar to those documented in wild plants. Across the five varieties evaluated here, high rates of photosynthesis (*A*) and leaf nitrogen (N) concentrations were coupled with low leaf mass per area (LMA), whereas the opposite suite of traits defined the “resource-conserving end” of this intraspecific LES in grape. Variety identity was the strongest predictor of leaf physiological (*A*) and morphological traits (i.e., leaf area and leaf mass), whereas leaf chemical traits and LMA were best explained by growth stage. All five varieties expressed greater resource-conserving trait syndromes (i.e., higher LMA, lower N, and lower *A*_mass_) later in the growing season. Traits related to leaf hydraulics, including instantaneous water-use efficiency (WUE), were unrelated to LES and other resource capture traits, and were better explained by spatial location. These results highlight the relative contributions of genetic, developmental, and phenotypic factors in structuring trait variation in the five wine grape varieties evaluated here, and point to a key role of domestication in governing trait relationships in the world’s crops.

## 1. Introduction

The Leaf Economics Spectrum (LES) represents a suite of six leaf functional traits—maximum photosynthetic assimilation (*A*) and dark respiration rates (*R*), leaf nitrogen (N) and phosphorus (P) concentrations, leaf mass per area (LMA), and leaf lifespan (LL)—which covary with one another across [1,2] and within [3,4] plant species. The LES trait syndromes expressed by species or individual plants in turn underpin plant resource-use or ecological strategies, which range from resource-acquiring strategies on one end of the LES, to resource-conserving strategies on the other [1,2,5]. In general, resource-acquiring species and plants express high rates of *A* and *R*, high leaf N, which are coupled with low LMA, and short LL; the opposite suite of traits reflects the resource-conserving end of the LES [2].

The LES trait syndromes of plants scale-up to influence different aspects of whole-plant physiology, form, and function [5,6]. For instance, species expressing resource-conserving LES traits or trait syndromes are commonly associated with shade-tolerant life-history strategies, whereas resource-acquiring species often represent early successional pioneer vegetation [7,8,9]. At the same time, LES traits also represent the mechanism by which plant diversity influences rates of ecosystem functioning. For example, certain LES traits, including leaf N, have been found to predict rates of leaf-litter decomposition and soil N availability [10], whereas other LES traits, including *A* and LMA, are central in vegetation dynamics models [11].

To date, much of the research on the ecological and evolutionary determinants of LES trait variation and relationships in plants has focused on LES trait expression in wild plants growing in unmanaged ecosystems [2,12]. However, more recently, studies have begun to quantify the extent, causes, and consequences of inter- and intraspecific LES trait variation in crops or their progenitors growing in managed systems. This includes studies on soy [13], coffee [14,15], wheat [16], maize [17], cocoa [18], rice [19], and sunflower [20], cultivated across field- and lab-based conditions. These studies have largely focused on: (1) quantifying how plants of the same crop species or variety differ from one another across the LES [15]; (2) elucidating the role environmental conditions, genetics, plant development, and/or domestication history plays in structuring LES trait variation in crops [18]; and finally, (3) assessing relationships between LES trait variation in crops and agroecosystem functions, including yield [13,21], tissue decomposition [22], soil microbial diversity [23], and plant–soil interactions such as N_2_ fixation [24].

Although results differ across studies and systems, some generalities have emerged from this line of research. First, most studies on crop LES trait variation have indicated that artificial selection has shifted certain crops towards expressing some of the most extreme resource-acquiring LES trait values observed among plants globally [17,25]. Second, multiple studies have reported that individual plants of the same crop species or genotype differ along an “infraspecific LES” (i.e., an LES that exists below the species level), which is largely driven by environmental conditions. Specifically, within a given crop, the resource-conserving end of an infraspecific LES is dictated by plants growing in unfavourable conditions (e.g., hot, dry, nutrient limited, and/or under soil compaction), whereas favourable growing conditions confer the expression of resource-acquiring LES trait syndromes [13,15].

Finally, research has consistently shown that the shape of infraspecific LESs (i.e., the slope of a bivariate statistical model that describes trait relationships) is both unique to a given crop, and often (but not always) differs from LES trait relationships observed among plants globally [13,15,19]. For example, compared with wild plants, coffee expresses lower *A* at a given leaf N concentration, which likely reflects the role selecting for non-photosynthetic N-based compounds (i.e., caffeine) plays in governing coffee LES trait relationships [14,15]. Alternatively, compared with wild plants, rice expresses higher rates of *A* for a given leaf N concentration, which likely reflects a history of artificial selection for improved N-use efficiency and growth [19]. However, other crops, including soy, exhibit relationships between *A* and leaf N that are statistically indistinguishable from those in wild plants [13]. Although certain generalities have emerged from the literature, multiple studies have indicated that infraspecific LES relationships are unique to individual crops. However, to date, there remain relatively few studies testing for the presence of LES trait relationships in crops, and evaluating whether these crop-specific LES trait relationships differ from a “universal LES” hypothesized to describe global plant trait variations.

This study evaluated LES trait relationships in wine grape (*Vitis vinifera* L.): one of the world’s most commercially important crops which, along with table grapes, is currently estimated to cover ~6.95 million ha of agricultural land globally. Considerable research on wine grapes to date has sought to quantify the extensive diversity in above- [26] and belowground functional traits [27], phenology [28,29], and physiognomic forms [26], that exists across the ~1100 varieties spanning multiple climatic zones [30]. More specifically, leaf physiological, chemical, and morphological trait variations have long been the focus of many studies in the areas of crop biology and viticulture [31]. However, to the best of our knowledge there have been no studies explicitly evaluating whether wine grapes vary along an infraspecific LES, or if the shape of an infraspecific wine grape LES differs from that observed among plants globally. This study aimed to fill this gap by quantifying nine LES and related leaf traits in five widely cultivated wine grape varieties (‘Chardonnay’, ‘Pinot Gris’, ‘Cabernet Sauvignon’, ‘Merlot’, and ‘Syrah’) at two growth stages (post-flowering and veraison). These data were then used to (1) quantify differences in LES traits across wine grape varieties and at different growth stages; (2) determine whether an infraspecific LES in wine grapes exists; and (3) test whether wine grapes differ from wild plants in their LES trait relationships.

## 2. Materials and Methods

### 2.1. Study Site and Design

This study was conducted at the Niagara College Teaching Vineyard (previously known as “Coyote’s Run” winery), situated in Niagara-on-the-Lake, Ontario, Canada (43.1697° N, 79.1193° W) (Figure S1). This vineyard is situated within the Lakeshore Plains Region in the Niagara Region, which is characterised by gentle slopes, lake-effect moderated temperatures, and high incident sunlight during the growing season. More specifically, based on downscaled climate data at a 1 km^2^ resolution [32], the study site experiences mean annual temperatures of 8.8 °C, receives mean annual precipitation rates of 895 mm year^−1^, is not irrigated, and is situated on top of sandy loam/red shale soils which are well drained. At the farm, common vineyard management systems are employed. This includes vines that are trained using a 2-arm flat vertical shoot position system, applications of calcium nitrate and/or muriate of potash and/or sulphate of potash magnesium (K-Mag; 22-10.8-22) applied uniformly across the farm in mid-June, and foliar spray of liquid calcium (8-0-0-10) is applied early in each growing season.

At the site, five of the most common grape varieties were selected for this study, including ‘Chardonnay’, ‘Cabernet Sauvignon’, ‘Merlot’, ‘Pinot Gris’, and ‘Syrah’. All vines were grafted on rootstock SO4 in 2004–2006. For each of these varieties, leaf traits were sampled on a total of nine plants, which were evenly distributed across three distinct sampling rows spaced ~10 m apart. Within each row, three individual vines were selected for assessments of leaf traits. Sampling rows and individual vines were marked with flagging tape to enable sampling at two different growth stages, including immediately following flowering or cap-fall (i.e., at approximately E-L stage number 25/26 [33]; 15–20 June 2021; hereafter, “post-flowering”), and during veraison (i.e., approximately at E-L stage number 36/37 [33]; August 10–15; hereafter, “veraison”). All vines chosen for this study were between 1 and 3 cm in basal diameter, and were free of major pest of pathogen damage. On each vine, one individual leaf was selected to perform detailed assessments of leaf traits. Leaves were all situated at ~1.5 m above ground, which corresponded to the top of each vine canopy. Leaves chosen for sampling were all recently developed, fully expanded, fully sun-exposed, and free of any signs of damage [34]. In summary, the trait dataset employed in this study included measurements of five varieties, with each variety being represented by nine vines, and each vine spaced across three planting rows (alternatively, 45 planting rows in total). Each vine was sampled at two growth stages for a total sample size of *n* = 90 leaves.

### 2.2. Functional Trait Measurements

For each leaf, nine physiological, morphological, and chemical traits were measured. In the field, an LI-6800 portable gas exchange analyzer (LI-COR Biosciences, Lincoln, Nebraska, USA) was used to evaluate leaf physiological traits, including maximum photosynthetic capacity on a per-leaf area basis (*A*_max_, μmol CO_2_ m^−2^ s^−1^), evapotranspiration rates (*E*, mmol H_2_O m^−2^ s^−1^), and stomatal conductance (*g*_s_, mol H_2_O m^−2^ s^−1^). All physiological measurements were taken before 13:00 to avoid stomatal closure, and under the following conditions: CO_2_ concentrations of 400 ppm, photosynthetic photon flux densities of 1500 μmol of photosynthetically active radiation (PAR) m^−2^ s^−1^, relative humidity at 53–74%, leaf vapour pressure deficits of 1.2–1.7 KPa, and leaf temperatures between 24.3 and 31.6 °C. All leaves were allowed to stabilize at these conditions for at least 5 min, prior to logging data, and values for these traits were calculated as the mean of three replicate measurements taken 20 s apart. Physiological trait data were also used to calculate instantaneous water-use efficiency (WUE, mmol CO_2_ mol H_2_O) as *A*_max_/*E*.

Once physiological measurements were completed, leaves were collected and transported to the University of Toronto Scarborough, Canada, for analyses of morphological and chemical traits. Here, leaves were first weighed for fresh leaf mass (g), and then an LI-3100C leaf area meter (LI-COR Biosciences, Lincoln, NE, USA) was used to measure leaf area (cm^2^). Subsequently, all leaves were dried at 60 °C to constant mass and re-weighed for dry mass (g). These data were used to calculate LMA (g m^−2^) as dry mass/fresh area, and LMA data were, in turn, used to derive mass-based maximum photosynthetic rates (*A*_mass_, mmol CO_2_ g^−1^ s^−1^) as *A*_max_/LMA. Finally, leaves were ground into a homogeneous fine tissue using an MM400 Retsch ball mill (Retsch Ltd., Hann, Germany), and ~0.1 mg of leaf tissue was weighed, placed into a foil capsule, and analysed for leaf N and C concentrations (both on a % dry mass) using a LECO CN 628 elemental analyzer (LECO Instruments, Ontario, Canada).

### 2.3. Data Analysis—Causes of Intraspecific Trait Variation in Wine Grape Varieties

All statistical analyses were performed using R v. 4.1.0 (R Foundations for Statistical Computing). The first analysis evaluated statistical distributions for all traits using the ‘*fitdist*’ function in the ‘*fitdistrplus*’ R package [35], to identify which traits were normally or log-normally distributed, as inferred by the highest log-likelihood values. Based on these results, descriptive statistics for each trait across our entire dataset (*n* = 90 observations for each trait) were calculated, which included means and standard deviations (SDs) for normally distributed traits, and medians and median absolute deviations (MADs) for log-normally distributed traits. Coefficients of variation (CVs) were also calculated for all traits.

An analysis of variance (ANOVA) procedure, coupled with Tukey’s honestly significant difference (HSD) post hoc tests, was then used to evaluate whether traits varied as a function of growth stage (i.e., post-flowering or veraison), variety identity, and planting row, as well as all two- and three-way interactions. This procedure was then paired with a variance partitioning analysis, employed in previous analyses of intraspecific trait variation [15,36], to identify the factors that explained the highest proportion of variability in grape traits. This entailed first fitting a linear mixed effects model with nested random effects using the ‘*lme*’ function in the ‘*nlme*’ R package [37]. In this model, nested random effects were parameterized as planting rows nested within varieties which were nested within the growth stage; a random intercept was included as the only fixed effects [36]. The ‘*varcomp*’ function in the ‘*ape*’ R package [38] was then used to partition the variance in a given trait across the nested random effects, while also quantifying the proportion of trait variability unexplained by the nested factors considered here.

### 2.4. Data Analysis—Bivariate and Multivariate Trait Correlations

Pearson correlation tests were used to evaluate all pairwise trait relationships across the entire dataset (*n* = 90 observations total for each test). Multivariate relationships among grape traits were then examined using a principal components analysis (PCA), which was implemented with the ‘*rda*’ in the ‘*vegan*’ R package [39]. In this PCA, all trait data were scaled to unit variance, and *A*_max_ was excluded due to its strong correlation with *A*_mass_ (*r* = 0.801, *p* < 0.001). The ‘*dimdesc*’ function in the ‘*FactoMineR*’ R package [40] was then used to evaluate the statistical relationships between individual traits and the first two principal component axes. Multivariate analysis also included a permutational multivariate analysis of variance (PerMANOVA), which was designed to test whether multivariate trait syndromes varied significantly as a function of planting row, variety, and growth stage, as well as all two- and three-way interactions among these factors. This PerMANOVA was performed using the ‘*adonis*’ function in the ‘*vegan*’ R package [39] and was based on *n* = 999 permutations. Finally, we used ANOVA coupled with a Tukey HSD post hoc test (implemented as above) to test whether PCA axis 1 and 2 scores varied as a function of growth stage, variety identity, and planting row, and all two- and three-way interactions.

### 2.5. Data Analysis—An Intraspecific LES across Wine Grape Varieties

The final statistical analysis evaluated relationships among three leaf traits which are central in the LES hypothesis, including LMA, *A*_mass_, and leaf N [2]. Here, standardized major axis (SMA) regression analysis was used to quantify pairwise trait relationships in grapes, and compare their shape (i.e., SMA slopes) and strength (i.e., SMA *r*^2^ values) with those same trait relationships observed among plants globally. This analysis entailed first fitting an SMA regression to the grape trait dataset (*n* = 90 leaves total) using the ‘*sma*’ function in the ‘*smatr*’ R package [41], and then performing this same analysis on plant species in the GLOPNET dataset of Wright et al. [2]. These GLOPNET analyses were based on *n* = 764 species with paired LMA-*A*_mass_ data, *n* = 1958 species with paired LMA-leaf N data, and *n* = 706 plant species with paired *A*_mass_-leaf N data. Finally, our analysis tested for statistically significant differences in the slopes of these LES trait relationships in grape vs. wild plants in GLOPNET, using the ‘*slope.test*’ function in the ‘*smatr*’ R package [41].

## 3. Results

### 3.1. Trait Variation across Wine Grape Varieties

**Figure 1:**
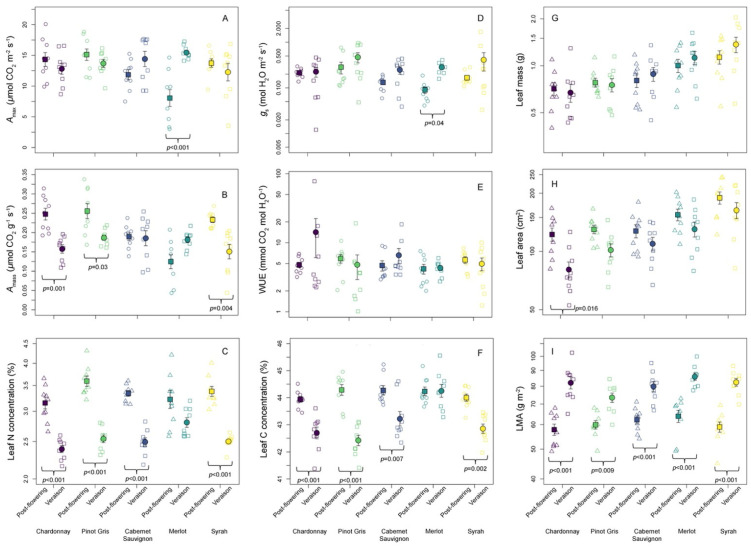
Functional trait variations across five wine grape varieties at two growth stages. Colours correspond to different wine grape varieties, with square symbols representing trait values measured post-flowering and circles representing trait values measured during veraison. Trait data are presented on log-scales where appropriate, as informed by the summary statistics presented in Table 1, and results from analyses of variance (ANOVAs), testing for differences in traits across varieties, growth stage, planting rows, and all interactions, are presented in Table 2. Additionally, certain results are presented from Tukey’s honestly significant difference (HSD) post hoc tests. For clarity, only instances where traits varied significantly within varieties across growth stages (Tukey’s HSD *p* < 0.05) are shown below a given contrast. Trait abbreviations are as follows: *A*_max_: light saturated maximum photosynthetic rate on a per-unit leaf area basis (Panel **A**); *A*_mass_: light saturated maximum photosynthetic rate on a per-unit leaf mass basis (Panel **B**); leaf N: leaf nitrogen concentration (Panel **C**); *g*_s_: stomatal conductance (Panel **D**); WUE: instantaneous water use efficiency (Panel **E**); leaf C: leaf carbon concentration (Panel **F**); leaf mass: leaf dry mass (Panel **G**); leaf area: fresh leaf area (Panel **H**); LMA: leaf mass per unit area (Panel **I**).

All traits ranged widely across the varieties and growth stages evaluated here, with all traits except LMA, leaf C, and leaf N expressing CVs ≥ 20 (Table 1). Physiological traits were particularly variable, such that *A*_max_ ranged from 2.320.1 μmol CO_2_ m^−2^ s^−1^ (CV = 26.7) and *A*_mass_ from 0.043–0.338 μmol CO_2_ g^−1^ s^−1^ (CV = 30.2). Similarly, WUE ranged widely from 1.0 to 19.1 mmol CO_2_ mol H_2_O^−1^ (CV = 63.9), and *g*_s_ ranged from 0.012 to 0.83 mol H_2_O m^−2^ s^−1^ (CV = 97.3). However, for these groups of traits, the factors best explaining this variability differed. Variation in both *A*_mass_ and *A*_max_ was best explained by grape variety identity (explained variance = 27.3% and 37.8%, respectively), whereas variation in WUE and *g*_s_ was best explained by spatial location/row identity (explained variance = 9.2% and 12.9%, respectively). Variation in traits related to leaf size, including leaf dry mass (range = 0.399–2.04 g) and leaf area (range = 52.6–241.7 cm^2^), was also best explained by variety identity (explained variance = 29.4% and 35.5%, respectively). Leaf chemical traits, including leaf C and N concentrations, were the least variable (CV = 2.0 and 17.0, respectively), with values ranging from 41.4% to 45.6% C and 2.2% to 4.3% N. Leaf chemical traits, along with LMA, were best explained by growth stage, thus reflecting trait variation that occurs as plants developed from post-flowering to veraison (Table 1, Figure 1).

**Figure 2:**
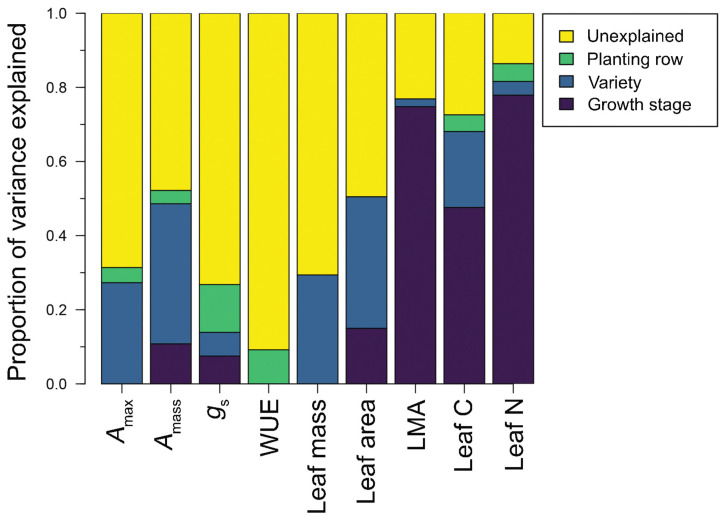
Variance partitioning of nine leaf traits measured across five common wine grape varieties, at two growth stages, and across spatial locations (i.e., planting rows). Heights of the different bar segments correspond to the proportion of total trait variation in each trait (where *n* = 90 leaves for all traits), as determined by a variance partitioning procedure. Variance components presented here visually correspond to the numerical values presented in Table 1. Trait abbreviations are as follows: *A*_max_: light saturated maximum photosynthetic rate on a per-unit leaf area basis; *A*_mass_: light saturated maximum photosynthetic rate on a per-unit leaf mass basis; *g*_s_: stomatal conductance; WUE: instantaneous water use efficiency; LMA: leaf mass per unit area.

With the exceptions of WUE and *g*_s_, variance partitioning and ANOVA indicated that variety identity, growth stage, and a variety-by-growth stage interaction term were the most important factors determining leaf trait variation in our dataset (Table 2). Across all traits except for WUE and *g*_s_, the combination of variety identity and growth stage explained between 27.3% and 81.5% of trait variation (Figure 2, Table 1). Moreover, except in the case of *A*_max_, traits varied significantly as a function of variety and growth stage (Figure 1 and Figure 2, Table 1). Across varieties, ‘Pinot Gris’ most consistently expressed a suite of traits that were the clearest ‘resource-acquiring’ syndrome. In the dataset, ‘Pinot Gris’ expressed among the highest values of *A*_max_ (15.1 and 13.7 μmol CO_2_ m^−2^ s^−1^ in the post-flowering and veraison stages, respectively), *A*_mass_ (0.256 and 0.186 μmol CO_2_ g^−1^ s^−1^ in the post-flowering and veraison stages, respectively), and leaf N (3.4% and 2.5% in the post-flowering and veraison stages, respectively), and the lowest LMA values (59.9 and 73.4 g m^−2^ in the post-flowering and veraison stages, respectively; Figure 1).

One of the most consistent patterns observed in this analysis is that across all varieties and traits, grapes generally express more ‘resource conservative” trait syndromes in their leaves as they develop from the post-flowering through to veraison stages. This entailed all varieties expressing statistically significant (Tukey’s HSD *p* < 0.05) increases in LMA between the post-flowering and veraison stages, four varieties expressing statistically significant (Tukey’s HSD *p* < 0.05) declines in leaf N, and three varieties expressing statistically significant (Tukey’s HSD *p* < 0.05) declines in *A*_mass_ over the same stages (Figure 1). Additionally, consistent with plants moving towards more resource-conserving trait syndromes through the growing season, leaves were smaller in area during veraison than post-flowering within all varieties, although these differences were not statistically significant (Figure 1). Four of the five varieties also expressed statistically significant declines (Tukey’s HSD *p* > 0.05) in leaf C concentrations between the two growth stages (Figure 1).

### 3.2. Relationships among LES and Other Leaf Traits in Wine Grape Varieties

**Figure 3:**
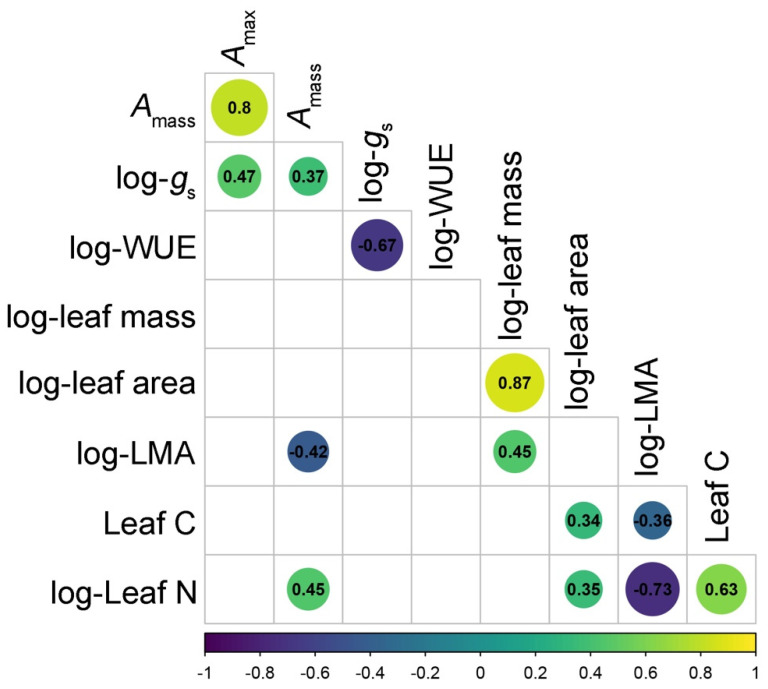
Pearson correlation tests analyzing the relationships between nine leaf traits measured across five wine grape varieties in Southern Ontario, Canada. Shades of circles correspond to Pearson correlation coefficients for each test which are presented numerically within the circles. Sample sizes for each correlation test were *n* = 90 leaves, and only statistically significant trait correlations represented by circles/correlation coefficients (where *p* ≤ 0.05) are presented here. A full trait correlation matrix is presented in Table S1. Trait abbreviations are as follows: *A*_max_: light saturated maximum photosynthetic rate on a per-unit leaf area basis; *A*_mass_: light saturated maximum photosynthetic rate on a per-unit leaf mass basis; *g*_s_: stomatal conductance; WUE: instantaneous water use efficiency; LMA: leaf mass per unit area.

Trait relationships in grape were largely consistent with patterns observed in the LES, including positive relationships among *A*_mass_ and leaf N (Pearson *p* < 0.001, *r* = 0.448), both of which traded-off with LMA (Pearson *r* = −0.424 and *r* = −0.727, respectively, *p* < 0.001 in both cases; Figure 3, Table S2). Leaf C concentrations also expressed significant relationships with certain LES traits; notably, a positive correlation with leaf N (Pearson *r* = 0.63, *p* < 0.001) and a negative relationship with LMA (Pearson *r* = −0.359, *p* < 0.001; Figure 3, Table S2). Traits associated with plant–water relationships were correlated with one another (Pearson *r* = −0.67, *p* < 0.001); however, WUE and *g*_s_ were unrelated to any other traits measured here associated with C assimilation, leaf chemistry, or leaf size (Figure 3, Table S2).

**Figure 4:**
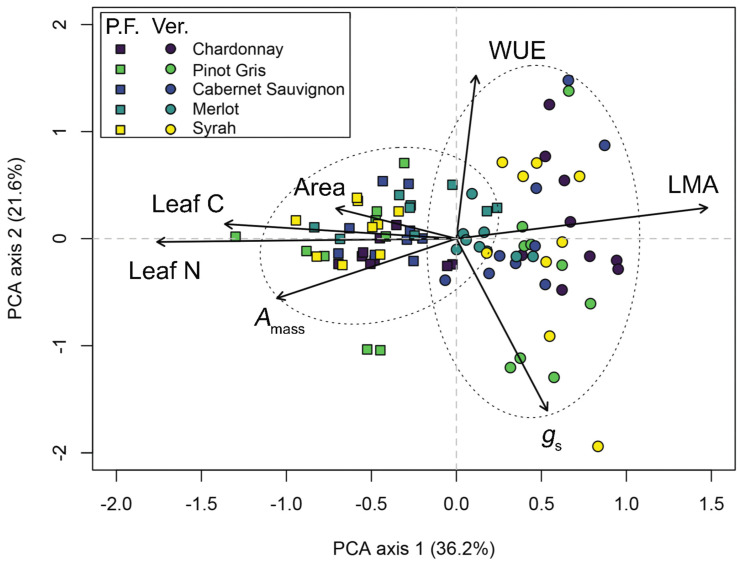
Principal components analysis (PCA) evaluating multivariate trait relationships across five wine grape varieties across two growth stages. Only seven of nine traits quantified in this study were included here, due to strong collinearity in certain traits (see Figure 3). Colours correspond to different varieties, whereas symbols represent different growth stages (post-flowering and veraison, “P.F.” and “Ver.”, respectively). To aid in visualization, also presented here are 95% confidence ellipses surrounding the two different growth stages which explained 22.9% of the variation in the seven traits analyzed here. Associated permutational analysis of variance (PerMANOVA) and relationships between individual traits and PCA axes are presented in Tables S2 and S3. Trait abbreviations are as follows: *A*_max_: light saturated maximum photosynthetic rate on a per-unit leaf area basis; *A*_mass_: light saturated maximum photosynthetic rate on a per-unit leaf mass basis; *g*_s_: stomatal conductance; WUE: instantaneous water use efficiency; LMA: leaf mass per unit area.

Multivariate analysis revealed that the first two principal components explained 36.2% and 21.6% of the trait variation in grape traits (Figure 4). Consistent with the results from bivariate analyses, the first PCA axis was significantly associated with LES traits, including *A*_mass_ (*r* = 0.559, *p* < 0.001), and leaf N (*r* = 0.932, *p* < 0.001), which trade off against LMA (*r* = −0.782, *p* < 0.001; Figure 4, Table S2). Other traits including leaf C (*r* = 0.721, *p* < 0.001) and leaf area (*r* = 0.375, *p* < 0.001) also loaded onto the first principal component axis, thereby contributing to the suite of traits that reflect resource acquisition (Figure 4, Table S2). The second principal component was primarily defined by WUE (*r* = 0.806, *p* < 0.001) which traded off against *g*_s_ (*r* = −0.851, *p* < 0.001; Figure 4, Table S2).

The PerMANOVA was consistent with univariate analyses of traits and causes of trait variation, with both growth stage and variety being statistically significant predictors of multivariate trait syndromes. These two factors explained a total of 50.3% of the variation in traits, with variety identity explaining 27.4% and growth stage explaining an additional 22.9% of variation (PerMANOVA *p* < 0.001 in both cases; Table S3). Although variety differences were less distinguished in our PCA, trait observations measured at different growth stages were clearly differentiated across the first PCA axis. Specifically, leaves from all varieties sampled in the post-flowering period were strongly associated with the resource-acquiring end of the first PCA axis, which reflected larger leaves with a higher *A*_mass_, leaf N, and leaf C, and a lower LMA. The opposite suite of traits characterized leaves from all varieties sampled during veraison (Figure 4). This finding was also confirmed by ANOVA, which revealed that PCA 1 axis scores varied significantly as a function of growth stage as well as a sampling time-by-variety interaction term (Table 2).

### 3.3. A Leaf Economics Spectrum across Wine Grape Varieties

**Figure 5:**
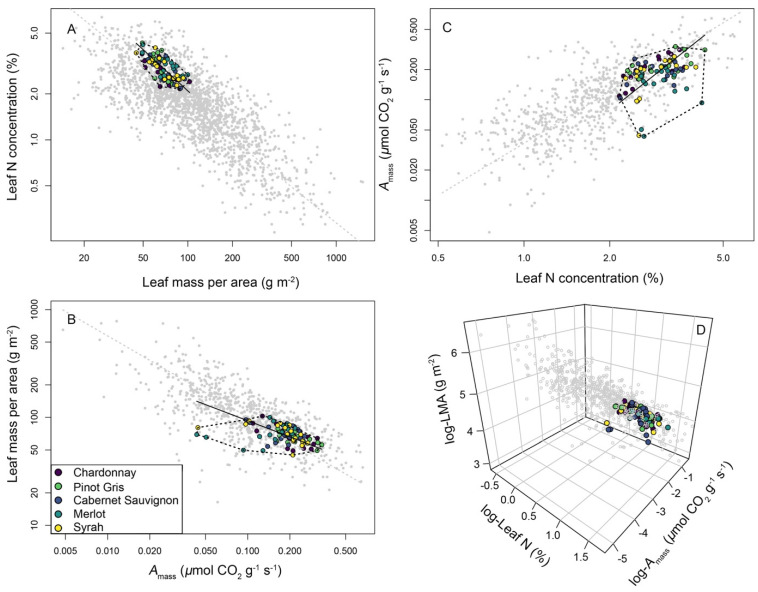
Leaf Economics Spectrum trait relationships in wine grapes. Presented here are bivariate relationships across three core LES traits including Amass, LMA, and leaf N concentrations (Panels **A**–**C**), as well as a three-dimensional representation of the relationships across the same traits (Panel **D**). Coloured points correspond to different grape varieties, which are not differentiated based on growth stage here to aid in visualization. Black solid trend lines correspond to the standardized major axis (SMA) regression model of a given bivariate trait relationship across wine grapes (where SMA model *p* < 0.05 and *r*^2^ ≥ 0.189 in all cases) and dashed black trend lines in (Panels **A**–**C**) represent convex hull models that encapsulate the two-dimensional trait space occupied by wine grape leaves. Additionally, data and SMA models for the same LES trait relationships observed among wild plants in the GLOPNET dataset are shown in all panels (grey dashed trend lines and points). SMA models were fit to the ‘Wine grape variety’ dataset, and wild plants were derived from the GLOPNET dataset (Wright et al., 2004). Full model diagnostics for each SMA model in (Panels **A**–**C**) are presented in Table S4. Trait abbreviations are as follows: *A*_mass_: light saturated maximum photosynthetic rate on a per-unit leaf mass basis; LMA: leaf mass per unit area.

Relationships among three core LES traits evaluated here including *A*_mass_, leaf N, and LMA, closely matched patterns of LES trait variation observed among plants globally. This included positive SMA relationships among *A*_mass_ and leaf N (SMA model *r*^2^ = 0.189, *p* < 0.001), and negative relationships between LMA and *A*_mass_ (SMA model *r*^2^ = 0.191, *p* < 0.001), and LMA and leaf N (SMA model *r*^2^ = 0.507, *p* < 0.001; Figure 5, Table S4). Positive scaling relationships between *A*_mass_ and leaf N in grape (SMA model slope = 0.12) were statistically indistinguishable from the *A*_mass_-leaf N observed in the GLOPNET dataset of plants globally (SMA model slope = 0.11, slope test *r* = 0.03, *p* = 0.77; Figure 5, Table S4). Analysis did identify statistically significant differences in LES trait scaling relationships between LMA and leaf N in grapes which were steeper (SMA model slope = −0.04) vs. the GLOPNET dataset (SMA model slope = −0.008; slope test *r* = 0.96, *p* < 0.001; Figure 5, Table S4). Similarly, relationships between *A*_mass_ and LMA in grapes (SMA model slope = −225.2) differed statistically from those observed in the GLOPNET dataset (SMA model slope = −868.1; slope test *r* = −868.1, *p* < 0.001; Figure 5, Table S4).

## 4. Discussion

This study reveals that the five wine grape varieties evaluated here differ significantly in their leaf physiological, chemical, and morphological traits. Specifically, analyses revealed that variety differences in *A*_mass_, leaf C, leaf N, leaf size (both mass and area), and, to a lesser extent, LMA, reflect differences in ecological strategies among varieties (Figure 4). Many prior studies have evaluated leaf trait variations in multiple *V*. *vinifera* varieties, across multiple environmental conditions and growth stages. Although a comprehensive review of these studies is beyond the scope of our analysis, these studies do suggest that our trait values broadly fall within the functional trait space occupied by wine grapes. For example, previous studies indicated the following trait ranges for vines of multiple varieties across a diverse set of conditions and stages: *A*_max_ between ~5 and 20 μmol CO_2_ m^−2^ s^−1^, leaf N between ~1.5% and 3.3%, and LMA ranging from ~30 to 270 g m^−2^ [42,43,44,45,46,47,48,49,50,51,52,53,54]. The dataset in this study broadly aligns with these findings, indicating that unlike other crops such as soy [13], wheat, and maize [17], which occupy the extreme “resource-acquiring” end of the LES trait space, wine grapes are largely intermediary in their LES trait values when compared with plants globally (Figure 5). The dataset here provides some evidence indicating that certain varieties (‘Chardonnay’ and ‘Pinot Gris’) express more resource-acquiring trait syndromes than others (‘Cabernet Sauvignon’, ‘Syrah’, and ‘Merlot’). This trend is intriguing and points to differences in red vs. white varieties; however, considerable overlap remains among varieties within the larger wine grape trait space (Figure 5), and our dataset contains too few varieties to support robust analyses of differences in trait syndromes across reds vs. whites.

Although variety identity explains up to 37.8% of leaf trait values (Table 1), changes in trait syndromes across growth stages were also a pronounced determinant of trait syndromes in wine grape. During veraison, across vines of all varieties leaves shift from resource-acquiring to resource-conserving leaf trait syndromes. With few exceptions, all varieties exhibited declines in *A*_max_, *A*_mass_, leaf N, and leaf area, in addition to increases in LMA between the two growth stages, and leaves were strongly and statistically differentiated in multivariate trait space according to growth stage (Figure 4). Taken together, variety differences, changes in traits related to vine development within a growing season, and their interactions were the most important factors structuring intraspecific leaf trait variations in grape. Alternatively, finer-scale spatial variation in traits within a given variety at a given sampling time—accounted for here as sampling row identity—explained little variation in leaf traits, particularly in the traits associated with the C economy of leaves.

Systematic varietal differences in longer-term leaf hydraulic traits, including water potential at turgor loss point, have been well documented, and indeed represent a primary basis of variety selection under climate change [55]. However, in this study, unlike traits reflecting hydraulic safety margins or resource capture traits, we found *g*_s_ and WUE did not strongly vary across varieties or sampling times. Instead, these traits were better explained by planting row variations, although systematic differences across rows were not statistically significant (Table 2). This finding is consistent with previous research on crop traits reporting that, at the farm scale, leaf hydraulic traits reflecting short-term water fluxes such as WUE are often better explained by localized environmental conditions [13].

The present study was performed over a single growing season. Therefore, the analysis here is limited in informing how longer-term climatic, edaphic, and/or management-related changes influence grape leaf trait expression. Crop responses to a multitude of chronic, acute, and interacting environmental drivers underpin longer-term agroecological resistance or resilience environmental change [56], with crop leaf [29,57], root [27], or phenological traits [28,58] being central in meditating these responses. Indeed, enhancing the phenological variability and other aspects of functional diversity has been identified as a key climate change mitigation strategy for vineyards globally [30]. Expanding the research here to understand how LES traits of different wine grape varieties respond to environmental variability—e.g., elevated temperatures, increases in atmospheric CO_2_ concentrations, or water limitation—therefore represents an avenue for understanding how vineyard functional diversity confers resistance or resilience to environmental change [12,43,59,60].

There was some evidence that grape falls along an infraspecific LES consistent with that observed in wild plants [1,2]. This includes statistically significant positive covariation between *A*_mass_ and leaf N, both of which trade off with LMA (Figure 5). This finding contributes to existing studies on intraspecific LES trait variation, showing that plants of multiple domesticated plant species differentiate along intraspecific Leaf Economics Spectra [13,15,19]. This also aligns with recent studies showing that within ‘Chardonnay’ alone, vines differ in their resource capture traits in response to soil compaction [61]. Here, this study found that in wine grape varieties, relationships between leaf N and *A*_mass_ are statistically indistinguishable from the same relationship observed in plants globally (Figure 5). Additionally, although other LES trait relationships in grape evaluated here including those between LMA, *A*_mass_, and leaf N, did differ statistically from those quantified in the GLOPNET dataset, grape LES traits and their relationships were broadly aligned in both datasets (Figure 5).

This is unlike certain crops, including rice [19] and coffee [15] which, due to their domestication syndromes that favour greater resource-use efficiencies or concentrations of secondary compounds, exhibit LES trait relationships that differ statistically from those observed in wild plants. Instead, wine grapes appear to align more closely with crops such as soy whose LES relationships match closely with wild plants [13]. When taken with the broader literature on crop traits, the following hypothesis emerges: LES trait relationships in crops differ from those in wild plants, when the domestication syndrome entails targeted alterations to the N economy of leaves and plants.

In the present study, the sample sizes for individual varieties (*n* = 18) were too small to evaluate whether varieties differed in their LES trait relationships [62]. However, one emerging question within studies on intraspecific leaf trait relationships, is whether or not plants are constrained along a single LES which is unique to a given species or genotype. To date, this has been only weakly addressed, with different datasets from coffee indicating that a single intraspecific LES describes plant trait syndrome differences across growing conditions [15] and ontogeny [14]. Therefore, expanding the study design here to include a greater number of wine grape varieties, and expanding sampling designs to include a wider range of environmental conditions and growth stages, would inform this question.

In defining the traits that form the LES in plants, this study also shows that in wine grapes, leaf C concentrations correlate positively with traits associated with resource acquisition, including leaf N and (in multivariate space) *A*_mass_, while trading off against LMA (Figure 3 and Figure 4). In other crops, leaf C has been found to reflect leaf construction costs, and therefore, positively correlate with LMA and leaf dry matter content; by extension, higher leaf C values generally reflect a more resource-conserving trait strategy [15,21]. However, here and in our previous research on ‘Chardonnay’ traits [61], higher leaf C values were associated with a more resource-acquiring trait syndrome. In addition to strong inter-varietal variation in leaf C, we detected consistent and pronounced seasonal declines in leaf C in all varieties except ‘Merlot’ (Figure 1). A coupling of leaf C concentrations with other LES traits (namely, leaf N) in wine grape leaves is likely as a result of commensurate changes in enzymes related to both the Calvin cycle and starch and sugar synthesis [63]. By extension, trait relationships that involve leaf C concentrations may reflect a component of a domestication syndrome in wine grapes, namely, artificial selection for starches and sugars in leaves which then deplete during veraison [31].

## 5. Conclusions

Our study contributes to the growing literature indicating that plants of the same crop species differ from one another in their leaf traits, with plants differentiating along infraspecific LESs that show important similarities and differences from the LES observed in plants globally. This study finds that variety and growth stage differences in leaf traits are most important in structuring trait-based ecological strategies of wine grapes, with fine-scale spatial variation being a smaller component. By extension, these findings also indicate that expanding this line of research to include additional wine grape varieties, along with multiple sites with contrasting environmental conditions, would likely be most important in expanding our understanding of the extent and drivers of LES trait variation in wine grapes. Indeed, although the five varieties included in the present study are widespread and common, they represent a small fraction of the total varietal diversity of wine grapes and growing conditions globally [30]. Despite this limitation to the present study, the analyses presented here indicates that significant genetic variation exists across wine grape varieties, as well as temporal variation in relation to plant development and reproduction. Better understanding (1) how the leaf traits studied here respond to environmental changes, (2) how these responses differ across varieties, and (3) the temporal sensitivity of trait-based responses to environmental fluctuations, represents key considerations for quantifying the role that functional diversity plays in mitigating climate change impacts on vineyards.

## Figures and Tables

**Table 1:** Descriptive statistics for nine leaf functional traits measured across five grape varieties at two different growth stages.

		Distribution Fitting		Descriptive Statistics				Variance Partitioning			
Trait Group	Trait	Normal	Log-Normal	Mean/ Median	SD/ MAD	Range	CV	Time	Variety	Row	Unexplained
Physiological	*A*_max_ (μmol CO_2_ m^−2^ s^−1^)	−240.4	−261.8	13.2	3.52	3.0–20.1	26.7	0.000	0.273	0.041	0.686
	*A*_mass_ (μmol CO_2_ g^−1^ s^−1^)	129.6	115.5	0.19	0.06	0.04–0.34	30.1	0.108	0.378	0.036	0.478
	*g*_s_ (mol H_2_O m^−2^ s^−1^)	−0.9	44.2	0.188	0.13	0.012–0.83	97.3	0.075	0.064	0.129	0.732
	WUE (mmol CO_2_ mol H_2_O^−1^)	−318.0	−222.5	4.3	2.2	1.02–19.1	63.9	0.000	0.000	0.109	0.891
Morphological	Leaf dry mass (g)	−32.8	−25.1	0.88	0.36	0.4–2.0	37.9	0.000	0.294	0.000	0.706
	Leaf area (cm^2^)	−464.7	−462.1	125.6	37.5	52.6–241.7	32.5	0.150	0.355	0.000	0.495
	LMA (g m^−2^)	−357.9	−357.2	68.8	14.6	44.9–102.8	18.4	0.748	0.021	0.000	0.231
Chemical	Carbon (% mass)	−116.8	−117.1	43.6	0.9	41.4–45.6	2.0	0.476	0.205	0.045	0.275
	Nitrogen (% mass)	−64.6	−61.6	2.9	0.5	2.2–4.3	17.0	0.779	0.037	0.048	0.136

Trait distributions were determined based on the highest log-likelihood. For normally distributed traits, means and standard deviations (SDs) are presented, whereas for log-normally distributed traits, median and median absolute deviations (MADs) are presented. Sample sizes in all cases are *n* = 90 leaves, and trait abbreviations are as follows: *A*_max_: light saturated maximum photosynthetic rate on a per-unit leaf area basis; *A*_mass_: light saturated maximum photosynthetic rate on a per-unit leaf mass basis; *g*_s_: stomatal conductance; WUE: instantaneous water use efficiency; LMA: leaf mass per unit area.

**Table 2:** Results of analysis of variance (ANOVA) testing variation in nine leaf traits and two principal components axis scores across two growth stages, five varieties, individual planting rows, as well as all two- and three-way interaction terms (denoted by “*”).

Trait Group	Trait	Growth Stage	Variety	Row	Stage * Variety	Stage * Row	Variety * Row	Stage * Variety * Row
Physiological	*A*_max_	3.18 (0.08)	1.92 (0.119)	0.64 (0.529)	**7.99** **(<0.001)**	0.08 (0.92)	1.04 (0.417)	1.73 (0.11)
	*A*_mass_	**18.49** **(<0.001)**	**6.46** **(<0.001)**	1.17 (0.317)	**10.22** **(<0.001)**	0.07 (0.933)	0.94 (0.495)	1.82 (0.091)
	log-*g*_s_	**6.95** **(0.011)**	1.96 (0.112)	2.1 (0.132)	**2.66** **(0.041)**	1.61 (0.208)	1.52 (0.17)	1.38 (0.225)
	log-WUE	0.05 (0.832)	1.795 (0.146)	0.773 (0.466)	0.368 (0.83)	0.92 (0.403)	2.78 (0.011)	0.45 (0.889)
Morphological	log-Dry mass	0.65 (0.425)	**8.17** **(<0.001)**	1.01 (0.369)	0.54 (0.705)	0.02 (0.984)	0.64 (0.744)	0.43 (0.897)
	log-Area	**18.72** **(<0.001)**	**12.39** **(<0.001)**	1.17 (0.318)	0.82 (0.52)	0.12 (0.885)	0.61 (0.77)	0.44 (0.895)
	log-LMA	**146.87** **(<0.001)**	2.26 (0.074)	0.26 (0.775)	1.38 (0.253)	0.12 (0.885)	1.23 (0.297)	1.11 (0.372)
Chemical	Leaf C	**86.13** **(<0.001)**	**9.26** **(<0.001)**	0.07 (0.937)	**7.15** **(<0.001)**	0.66 (0.521)	2.02 (0.059)	1.53 (0.168)
	log-Leaf N	**261.85** **(<0.001)**	**4.2** **(0.005)**	0.82 (0.45)	**4.79** **(0.002)**	1.5 (0.232)	**2.76** **(0.012)**	1.83 (0.089)
Multivariate	PCA 1	**374.9** **(<0.001)**	**2.26** **(0.073)**	0.627 (0.537)	**8.477** **(<0.001)**	0.561 (0.574)	1.56 (0.156)	1.72 (0.112)
	PCA 2	0.252 (0.617)	1.407 (0.243)	0.904 (0.41)	0.953 (0.44)	1.118 (0.334)	1.604 (0.143)	1.119 (0.364)

Values shown here are *F*-statistics and associated *p*-values (in parentheses), where the sample size for all ANOVAs was *n* = 90 leaves distributed equally across two growth stages (*n* = 45 leaves per growth stage total), five varieties (*n* = 18 leaves per variety), and three rows for each growth stage-by-variety combination. Statistically significant effects (where *p* < 0.05) are highlighted in bold, and abbreviations are as follows: *A*_max_: light saturated maximum photosynthetic rate on a per-unit leaf area basis; *A*_mass_: light saturated maximum photosynthetic rate on a per-unit leaf mass basis; *g*_s_: stomatal conductance; WUE: instantaneous water use efficiency; LMA: leaf mass per unit area; PCA 1: principal component 1 score; PCA 2: principal component 2 score. Results and associated post hoc tests for individual traits are presented in Figure 1.

## Data Availability

Data are not yet provided, but will be archived in the TRY Functional Trait Database upon publication of the manuscript.

## Supplementary Materials

The following supporting information can be downloaded at: https://www.mdpi.com/article/10.3390/plants11202792/s1, Table S1. Results of Pearson correlation tests evaluating bivariate relationships across nine leaf traits measured across five wine grape varieties in Southern Ontario, Canada, at two growth stages. Table S2. Contributions of leaf traits towards two primary axes in a principal component analysis (PCA) across wine grape leaves, measured on five varieties at two growth stages during the growing season. Table S3. Results of a permutational multivariate analysis of variance (PerMANOVA) evaluating variations in seven leaf traits measured in *n* = 90 leaves from five different grape varieties at two growth stages. Table S4. Standardized major axis (SMA) regression models evaluating bivariate correlations in three traits forming the Leaf Economics Spectrum (LES). Figure S1. Location of the Niagara College Teaching Vineyard, situated in Niagara-on-the-Lake, Ontario, Canada (43.1697° N, 79.1193° W).
