# Supplementary material for: Intraspecific Leaf Trait Variation across and within Five Common Wine Grape Varieties
Source: Plants (Basel). 2022 Oct 21;11(20):2792. doi: 10.3390/plants11202792 (PMC9611564; doi:10.3390/plants11202792)
Supplement: Supplementary file 1 [file plants-11-02792-s001.zip › plants-1915033-supplementary.pdf]

Supplemental Information for:

Research Article

Title: Intraspecific Leaf Trait Variation Across and Within Five Common Wine Grape Varieties

Authors Information: Samantha C. Macklin [1], Rachel O. Mariani [1], Emily N. Young [1], Rosalyn Kish [1], Kimberley A. Cathline [2], Gavin Robertson [2] and Adam R. Martin [1,*]

1 Department of Physical and Environmental Sciences, University of Toronto Scarborough, Toronto, ON M1C 1A4, Canada

2 Horticultural & Environmental Sciences Innovation Centre, Niagara College, Niagara-on-the-Lake, ON L0S 1J0, Canada

* Correspondence: adam.martin@utoronto.ca

Table S1. Results of Pearson correlation tests evaluating bivariate relationships across nine leaf traits measured across five wine grape varieties in Southern Ontario, Canada, at two growth stages. Sample sizes for each correlation test were *n*=90 leaves. Statistically significant trait correlations (where $p \leq 0.05$) are highlighted in bold and are also presented visually in Figure 3 of the main text.

	A_{max}	A_{mass}	log-g_s	log-WUE	log-Leaf mass	log-Leaf area	log-LMA	Leaf C	log-Leaf N
A_{max}	–	**<0.001**	**<0.001**	0.16	0.202	0.621	0.093	0.68	0.779
A_{mass}	0.801	–	**<0.001**	0.409	0.234	0.374	**<0.001**	0.11	**<0.001**
log-g_s	0.469	0.366	–	**<0.001**	0.97	0.614	0.326	0.194	0.245
log-WUE	0.149	0.088	-0.67	–	0.438	0.737	0.339	0.81	0.921
log-Leaf mass	0.136	-0.127	0.004	0.083	–	**<0.001**	**<0.001**	0.25	0.666
log-Leaf area	0.053	0.095	-0.054	0.036	0.868	–	0.654	**0.001**	**0.001**
log-LMA	0.178	-0.425	0.105	0.102	0.455	-0.048	–	**0.001**	**<0.001**
Leaf C	-0.044	0.17	-0.138	-0.026	0.122	0.338	-0.359	–	**<0.001**
log-Leaf N	0.03	0.448	-0.124	0.011	-0.05	0.354	-0.727	0.63	–

Table S2. Contributions of leaf traits towards two primary axes in a principal component analysis (PCA) across wine grape leaves, measured on five varieties at two different growth stages during the growing season. Correlation coefficients and associated *p*-values are derived from the '*dimdesc*' function in the '*FactoMineR*' R package. Only traits significantly contributing to each PCA axis (where $p<0.05$) are shown. Trait acronyms and units are defined and presented in Table 1 of the main text, and the PCA related to this analysis is presented visually in Figure 4 of the main text, with an associated permutational multivariate analysis of variance presented in Table S3.

**PCA Axis**	**Trait**	**Correlation**	***p*-value**
Axis 1	Leaf N	0.932	<0.001
	Leaf N	0.721	<0.001
	A_{mass}	0.559	<0.001
	Leaf area	0.375	<0.001
	g_s	-0.283	<0.001
	LMA	-0.782	<0.001
Axis 2	WUE	0.806	<0.001
	A_{mass}	-0.295	<0.001
	g_s	-0.851	<0.001

Table S3. Results of a permutational multivariate analysis of variance (PerMANOVA) evaluating variation in seven leaf traits measured in n=90 leaves from five different grape varieties at two growth stages in the growing season. The principal component analysis related to this PerMANOVA is presented visually in Figure 4 of the main text, and its statistically significant factor loadings are presented in Table S2.

**Factor**	**D.F.**	**Sums Of Sqs.**	**Mean Sqs.**	***F* value**	r^2	***p* value**
Growth stage	1	0.23	0.23	33.6	0.229	0.001
Variety	4	0.28	0.07	10.05	0.274	0.001
Row	2	0.01	0.01	0.76	0.01	0.558
Growth stage* variety	4	0.01	0.002	0.22	0.006	0.976
Growth stage* row	2	0.002	0.001	0.15	0.002	0.95
Variety * row	8	0.03	0.004	0.52	0.028	0.925
Growth stage* variety * row	8	0.04	0.02	0.74	0.04	0.728
Residuals	60	0.41	0.01		0.409	
Total	89	1.01			1	

Table S4. Standardized major axis (SMA) regression models evaluating bivariate correlations in three traits forming the Leaf Economics Spectrum (LES). Relationships among LES traits are presented here for grape (based on n=90 leaves measured across five varieties) and the GLOPNET dataset, which represents LES trait relationships across wild plants globally (where n=764 species used to evaluate the LMA-A_{mass} relationship, n=1,958 species for the LMA-leaf N relationship, and n=706 species for the A_{mass}-leaf N relationship). Also shown here are statistical tests evaluating differences in the slope of these LES relationships in grape vs. the GLOPNET dataset (where p<0.05 indicates a statistically significant difference in the slopes of the LES trait relationships between these two groups). Model diagnostics presented here are associated with the scatterplots presented visually in Figure 5 of the main text.

**LES Traits**		**Grape LES Models**				**GLOPNET LES Models**				**Slope tests**	
**X**	**Y**	**Intercept**	**Slope**	***p***	r^2	**Intercept**	**Slope**	***p***	r^2	***r* statistic**	***p***
A_{mass}	LMA	113.6	-225.2	<0.001	0.191	235.9	-868.1	<0.001	0.237	-868.1	<0.001
LMA	Leaf N	5.66	-0.04	<0.001	0.507	3.01	-0.008	<0.001	0.312	0.96	<0.001
Leaf N	A_{mass}	-0.15	0.12	<0.001	0.189	-0.09	0.11	<0.001	0.55	0.03	0.77

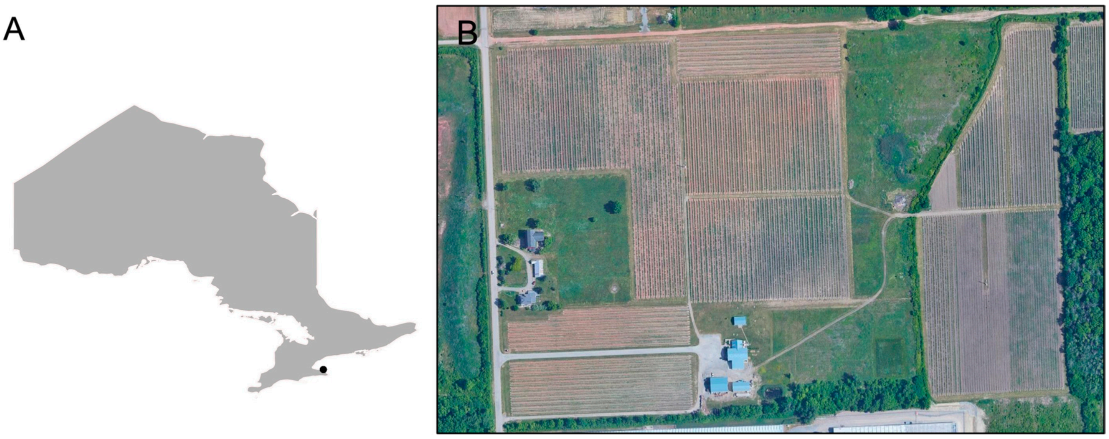

Figure S1. Location of the Niagara College Teaching Vineyard, situated in Niagara-on-the-Lake, Ontario, Canada (43.1697 °N, 79.1193 °W).
